# Physiotherapy for Patients with Sciatica Awaiting Lumbar Micro‐discectomy Surgery: A Nested, Qualitative Study of Patients' Views and Experiences

**DOI:** 10.1002/pri.1665

**Published:** 2016-02-23

**Authors:** Jonathan Boote, Ruth Newsome, Michael Reddington, Ashley Cole, Munyaradzi Dimairo

**Affiliations:** ^1^ University of Hertfordshire Hatfield UK; ^2^ University of Sheffield Sheffield UK; ^3^ Sheffield Teaching Hospitals NHS Foundation Trust Sheffield UK

**Keywords:** physical therapy modalities, qualitative research, sciatica

## Abstract

**Background and Purpose:**

Sciatica is a common clinical condition that can be extremely painful, disabling and life‐changing. Whether conservative or surgical treatment for sciatica secondary to an intervertebral disc prolapse is most effective is still much debated. An important component of conservative treatment is physiotherapy, which aims to promote physical and psychological health for the patient, whilst resorption of the disc takes place. This paper reports a qualitative study of patients' views and experiences of a bespoke physiotherapy intervention for the treatment of sciatica.

**Methods:**

A qualitative study nested within a pilot randomized controlled trial of bespoke physiotherapy for the treatment of patients with sciatica awaiting lumbar microdiscectomy surgery. Patients randomized to receive bespoke physiotherapy in the intervention arm of the trial were invited to take part in semi‐structured interviews. Twenty‐one in‐depth, semi‐structured interviews took place. All interviews were recorded, fully transcribed and thematically analysed.

**Results:**

Most patients in the sample found the physiotherapy valuable, appreciating the individual nature of the approach, the exercises to reduce pain and discomfort, techniques for improving functional spinal movement, walking and dynamic posture, and manual therapy and cardiovascular exercise. A small number did not find the physiotherapy of benefit. Sixteen patients in the sample went on to proceed with surgery, but most of these found value in having had the physiotherapy first.

**Discussion:**

Many patients with sciatica appreciate the value of physiotherapy prior to surgery. Future research should examine patients' experiences of bespoke physiotherapy delivered within primary care. Copyright © 2016 The Authors *Physiotherapy Research International* published by John Wiley & Sons Ltd.

## Introduction

Sciatica is a painful, often disabling condition and can be a major cause of disability, work loss and presentation to health‐care (Andersson, 1999; Waddell, 2004; Webster *et al.,* 2005). Sciatica is defined as leg pain in an area served by one or more spinal nerve roots and can be accompanied by neurological deficit such as altered power, sensation and/or reflexes (Koes *et al.,* 2007). Whether conservative or surgical treatment for sciatica is most effective is still much debated. It has been suggested that surgery generates improved outcomes in the short‐term but at 1 year, outcomes are very similar in both physiotherapy and surgical groups (Koebbe *et al.,* 2002; Luijsterburg *et al.,* 2007; Thomas *et al.,* 2007; Peul *et al.,* 2008; Weinstein *et al.,* 2008).

Although conservative treatment for sciatica has been advocated, there is little consensus on the most effective type and duration, or on the necessary level of experience of the practitioner delivering the treatment (Koes *et al.,* 2007; Luijsterburg *et al.,* 2007; Thomas *et al.,* 2007). It is not surprising therefore that the effectiveness of conservative treatment has shown wide variation (Vroomen *et al.,* 2000). A mainstay of conservative care in the UK is physiotherapy, which aims to promote physical and psychological health for the patient suffering with sciatica.

The natural history of sciatica caused by an intervertebral disc prolapse is unquestionably good. In light of evidence to suggest that spontaneous resorption of the disc fragment occurs (Autio *et al.,* 2006), physiotherapy allows the patient to manage their symptoms, whilst resorption takes place. Current guidelines for the management of back pain and sciatica reflect the evidence for the natural history of resolution of disc prolapse (European Commission Research Directorate General, 2004; Webster *et al.,* 2005; Haswell *et al.,* 2008). Further research is required in order to identify patient groups who respond well to conservative treatment methods.

This paper reports the findings of the qualitative component of a pilot trial of a physiotherapy intervention for people experiencing sciatica. The qualitative study was nested within a pilot randomized controlled trial (RCT) of physiotherapy for the treatment of patients with sciatica awaiting lumbar micro‐discectomy surgery. Details of the pilot trial itself can be viewed on the UK Clinical Research Network portfolio (UK Clinical Research Network: Portfolio Database, 2014). A qualitative component was included in the pilot RCT to enable patients to express their views, experiences and feelings about all aspects of the physiotherapy intervention and clinical pathway that they found helpful and unhelpful (Sheffield back pain, 2014).

## Methodology and methods

### Methodology

This qualitative study was informed by a constructivist methodology (Rodwell, 1998), with the intention of creating a joint understanding of each participant's views and experiences of the bespoke physiotherapy intervention that they had received. Constructivism assumes that the meaning of experiences and events are constructed by individuals and therefore, people construct the realities in which they participate (Charmaz, 2006). From this perspective, constructivist research aims to elicit and understand how research participants construct their meanings around the phenomenon of interest. Also particular to constructivism is a similar ‘construction of meaning’ by researchers that ‘their interpretation of the studied phenomenon is itself a construction’ (Charmaz, 2006, p. 187).

Within constructivist research, interviewing is a creative process in which the interactions and conversations of interviewer and respondent produce statements and formulations that draw upon the knowledge and experience of both participants. The co‐construction of participants' experiences by the researcher demands that qualitative constructivist research is a reflective and transparent process, where assumptions are articulated, and reflective, and analytical memos are written during the data collection and analysis process, in order to achieve transparency and rigour (Mills *et al.,* 2006). Such a constructionist qualitative framework seemed appropriate in a study attempting to describe views and experiences of a health‐care intervention.

### Interview process and topics discussed

All patients in the trial's physiotherapy (intervention) arm were invited to participate in an in‐depth interview to explore their views and experiences of the bespoke physiotherapy intervention they had received. The study received a favourable ethical opinion from the Sheffield Research Ethics Committee, and permission to proceed from the National Health Service Trust where the pilot trial took place. All patient participants signed a consent form before their involvement in the study. An additional consent form was used for all patients interviewed for the qualitative component.

Each interview was scheduled 2 weeks after the end of the physiotherapy programme for those not electing to have surgery, or up to 2 weeks post‐surgery for those who underwent surgery. The initial interview schedule was developed based on a review of the literature and refined following discussions with the study advisory group. Data collection and data analysis were inductive, whereby emergent issues arising from one interview were incorporated into the interview schedule of the next interview. All interviews were undertaken by J. B., a non‐clinical health services researcher with much experience of qualitative methods. All interviews were digitally recorded and transcribed verbatim, and a copy of the transcript was sent to each interviewee for their records, and for them to check for accuracy if they so wished. (The research team did not receive any corrections to any of the transcripts from the research participants.)

Topics for the interviews included the following: history of sciatica, its impact on quality of life, expectations and goals in having the physiotherapy, experiences of the referral pathway, physiotherapy goal fulfilment, anxieties at the start of the physiotherapy, degree of optimism at the start of the physiotherapy, perceived helpful and unhelpful aspects of the physiotherapy, the nature of the physiotherapy intervention, thoughts on any previous physiotherapy, concerns or anxieties prior to surgery and feelings about future health having undergone the physiotherapy. For those patients not proceeding with surgery, we asked their reasons for not having the surgery. For those patients proceeding with the surgery, we asked about their perceptions of having undergone the physiotherapy first, thoughts on the success of the surgery and the perceived value of post‐surgery physiotherapy. The topic guide used in the study can be viewed in Appendix 1 of the Supporting information.

### Description of the bespoke physiotherapy intervention

The physiotherapy intervention, discussed during the interviews, was delivered by one of two physiotherapists, who each received training delivering the intervention. An evidence‐based framework for assessment and treatment was developed to encompass the principle aspects, which occur in back pain and sciatica according to the literature. This framework was chosen as it enabled the treatment to be instigated by any physiotherapist with sound spinal knowledge and allowed for tailoring of the physiotherapy to individual patients' requirements. An example of how this approach was operationalized can be found in Appendix 2 of the Supporting information. All patients in the intervention arm of the trial received up to six sessions of physiotherapy.

### Recruitment process

The sample for the qualitative study was drawn from participants in the intervention arm of the pilot RCT who were listed for primary single level lumbar micro‐discectomy. During the recruitment process for the pilot trial, participants in the intervention arm were informed that they would be invited to participate in an in‐depth interview at the end of the physiotherapy intervention. Once each participant in the intervention arm had completed their course of physiotherapy, they were contacted by telephone to ascertain whether they were still content to take part in an interview. For those content to proceed, an interview was scheduled, with interviews taking place at the participant's own home, or at the clinic after the participant's physiotherapy appointment.

### Data management and approach to analysis

All interview transcripts were imported into the nvivo® data management system, and a thematic analysis was undertaken by two of the authors (J. B. — a health services researcher and R. N. — a clinical researcher and physiotherapist). The coding framework for the analysis was jointly agreed. J. B. coded all of the transcripts, and R. N. coded 25% for quality assurance purposes. The constant comparative method was utilized during data analysis, which allows themes to emerge from the codes and the way they are categorized (Boeije, 2002). Analysis of codes and emerging themes was undertaken as a team, enabling inter‐subjective validation through comparisons, and thus ensuring that consistency and robustness of the analysis were achieved (Charmaz, 2006). Each theme was given a code and examined in detail in terms of its analytical properties, and patterns were identified to formulate an emergent theme. Through further comparisons between codes, the key themes were identified.

### Ensuring rigour in the research process

The research team used a variety of strategies to ensure the rigour of the research (Tong et al., 2007), and these were particularly influenced by the constructivist methodology. In an effort to reduce bias and to demonstrate equipoise, the researcher who collected the qualitative data (J. B.) was not a physiotherapist. The emergent approach to data collection and analysis allowed us to establish when data saturation was reached. Member checking was utilized whereby respondents were given the opportunity to correct any factual inaccuracies in their interview transcripts. An audit trail of the analysis process was made possible by the use of nvivo® software. Finally, two members of the research team were involved in the data analysis process, in an effort to both enhance the trustworthiness of the findings and place the findings within a clinical context.

## Results

### Description of the sample

The demographic characteristics of patients in both arms of the pilot trial are contained in Table 1.

**Table 1:** Demographic details of participants

Variable	Scoring	Standard care	Physiotherapy	Total
		(*n* = 30)	(*n* = 29)	(*n* = 59)
Gender	Male	15 (50%)	14 (48%)	29 (49%)
	Female	15 (50%)	15 (52%)	30 (51%)
Ethnicity	British	29 (97%)	26 (90%)	55 (93%)
	Other	1 (0%)	3 (10%)	7 (8%)
Age (years)	*N* (%)	30 (100%)	29 (100%)	59 (100%)
	Mean (SD)	36 (7.4)	38 (6.0)	37 (6.7)
	Median (IQR)	37 (31 to 42)	37 (33 to 43)	37 (32 to 42)
	Min to max	19 to 48	26 to 50	19 to 50
Body mass index (kg·m^−2^)	*N* (%)	26 (87%)	29 (100%)	55 (93%)
	Mean (SD)	25 (3.8)	28 (4.4)	27 (4.3)
	Median (IQR)	25 (23 to 28)	27 (26 to 31)	26 (24 to 29)
	Min to max	19 to 34	19 to 38	19 to 38
Employment status	No	7 (23%)	9 (31%)	16 (27%)
	Full or part‐time	23 (77%)	20 (69%)	43 (73%)
Smoking status	Current smoker	12 (40%)	8 (28%)	20 (34%)
	Past smoker	1 (3%)	0 (0%)	1 (2%)
	Never smoker	16 (53%)	21 (72%)	37 (63%)
	Unknown	1 (3%)	0 (0%)	1 (2%)
Cigarette pack years	*N* (%)	13 (43%)	8 (28%)	21 (36%)
	Mean (SD)	9 (7.1)	13 (5.5)	11 (6.6)
	Median (IQR)	10 (4 to 13)	10 (8 to 19)	10 (8 to 17)
	Min to max	1 to 20	7 to 20	1 to 20
Diabetes status	No	29 (97%)	29 (100%)	58 (98%)
	Unknown	1 (3%)	0 (0%)	1 (2%)
Duration of symptoms				
(weeks)	*N* (%)	30 (100%)	29 (100%)	59 (100%)
	Mean (SD)	46 (46.3)	51 (42.8)	48 (44.3)
	Median (IQR)	29 (26 to 52)	32 (26 to 52)	32 (26 to 52)
	Min to max	9 to 260	12 to 208	9 to 260
	Severe	12 (40%)	12 (41%)	24 (41%)
	Crippled	8 (27%)	7 (24%)	15 (25%)
Oswestry Disability Index (%)	*N* (%)	30 (100%)	29 (100%)	59 (100%)
	Mean (SD)	48 (14.7)	48 (14.9)	48 (14.7)
	Median (IQR)	44 (36 to 64)	46 (34 to 60)	44 (36 to 60)
	Min to max	28 to 78	22 to 74	22 to 78
VAS back pain score	*N* (%)	30 (100%)	29 (100%)	59 (100%)
	Mean (SD)	51 (28.6)	57 (26.2)	54 (27.4)
	Median (IQR)	50 (27 to 73)	60 (40 to 78)	60 (31 to 78)
	Min to max	0 to 95	0 to 95	0 to 95
VAS leg pain score	*N* (%)	30 (100%)	29 (100%)	59 (100%)
	Mean (SD)	67 (26.1)	67 (20.0)	67 (23.1)
	Median (IQR)	70 (60 to 85)	69 (57 to 82)	70 (57 to 85)
	Min to max	5 to 100	20 to 98	5 to 100

BMI, body mass index; SD, standard deviation; IQR, inter‐quartile range.

Of the 24 patients in the intervention arm that were eligible to be interviewed, having completed their course of physiotherapy, 21 patients agreed to participate. Three patients were unable to be contacted by telephone. Of these 21 patients, 16 underwent surgery after their course of physiotherapy, whilst five decided not to proceed with surgery. The sample included nine men and 12 women, the age range of interviewees was 26–49 and the ethnic origin of most interviewees was White British. The demographic characteristics of the participants of the qualitative study are displayed in Table 2.

**Table 2:** Demographic characteristics of the patient sample

	ID No	Age	Gender	Ethnicity	ODI^1^ baseline	ODI^1^ follow‐up	VAS^2^ baseline back/leg	VAS^2^ follow‐up back/leg	Number of physiotherapy sessions	Surgery
1	1001	36	Male	White British	46	24	75/20	70/80	6	Yes
2	1004	33	Female	White British	36	40	60/94	98/80	4	Yes
3	1007	40	Female	White British	56	51	0/63	0/65	5	No
4	1008	41	Male	White British	40	24	30/41	21/52	6	Yes
5	1011	34	Female	White British	34	40	40/80	50/49	6	Yes
6	1014	45	Female	White British	74	62	39/67	38/60	2	Yes
7	1016	34	Female	Iranian/Kurdish	53	44	60/70	45/80	6	No
8	1019	31	Male	White British	44	18	55/65	4/5	6	No
9	1021	26	Female	Pakistani	60	56	95/98	91/94	6	No
10	1023	31	Male	White British	32	28	61/33	67/29	6	Yes
11	1025	36	Male	White British	68	60	86/92	82/85	3	Yes
12	1029	47	Male	White British	30	22	43/41	12/0	4	Yes
13	1030	36	Female	White British	46	40	81/78	71/65	6	Yes
14	1032	43	Female	White British	30	22	35/42	40/45	5	Yes
15	1036	39	Male	White British	46	50	78/60	54/37	5	Yes
16	1042	29	Female	Pakistani	66	42	80/44	12/12	6	No
17	1043	41	Male	White British	32	52	82/92	80/80	6	Yes
18	1045	49	Male	White British	30	24	45/57	10/37	6	Yes
19	1046	45	Female	White British	50	52	56/55	54/56	2	Yes
20	1049	30	Female	White British	60	2	3/72	0/0	6	Yes
21	1058	42	Female	White British	42	40	5/82	58/56	6	Yes

1 Oswestry Disability Index (0–100).

2 Pain Visual Analogue Scale (0–100).

The main codes for the patient interviews were as follows: history of the condition and previous treatment/personal stories, impact of sciatica on quality of life, referral by and interactions with their general practitioner, expectations/goals of having physiotherapy, anxieties/concerns/optimism prior to physiotherapy, reflections on the content of the physiotherapy received, helpful and less helpful aspects of the physiotherapy, concerns/anxieties prior to surgery, surgery/physiotherapy as joint interventions, current/future concerns about sciatica and degree of optimism about the future. From these codes, three main themes were derived: impact of sciatica on patients' quality of life, patients' expectations and perceptions of the bespoke physiotherapy intervention and patients' perceptions of the value of physiotherapy as an adjunct to surgery.

### Presentation of main themes

The three main themes that emerged during the data analysis process are presented in turn. Quotes from the patient interviews are employed to illustrate the analysis, with each quote accompanied by a unique identifier (in parentheses) in order to maintain participant anonymity. The unique identifier was the number allocated to the patient when they entered the pilot trial.

#### Impact of sciatica on patients' quality of life before they entered the pilot trial

Patients interviewed in this study spoke of how sciatica had disrupted their lives prior to entering the pilot trial. Most reported that the condition made moving difficult and painful, causing them to struggle with activities of daily living. *It started to affect my mobility, meaning that I couldn't bend as much. I certainly couldn't touch my toes any more, which therefore meant I couldn't put my knickers on in a morning properly, or put my socks on* (1032). Some also reported having to take sick leave from work: *at its worst, it resulted in me taking three weeks off work, sick* (1019).

Many patients reported that sciatica prevented them from participating in leisure activities. *It has had a big impact because I had to cut down on all my fitness. I couldn't go running. I had to stop playing football. I was in a lot of agony* (1043); *it restricted me running round with the kids in the garden* (1001). Some reported that the sciatica disrupted their sleeping patterns, whilst others reported that it led to anxiety, depression and a strain on family relationships*: It made me quite down and tearful, just mentally it took away my independence really. You are anxious, you are down. You take it out on people.* (1049). Others reported that there were times when the sciatica left them completely immobile, and reliant on analgesics*: Well at its worst, it has stopped me doing everything actually. All you can do is lie there basically. Take a lot of pain killers* (1023).

#### Patients' expectations and perceptions of the bespoke physiotherapy intervention

Patients reported a range of expectations about the physiotherapy prior to their treatment, including obtaining advice on managing the pain, improving mobility and flexibility and, for a small number in the sample, the hope that it might prevent the need for surgery: *My thoughts were that it would assist my movement* (1001); *I thought it would ease the pain a fair bit and also possibly relieve it enough so that I didn't need to have surgery* (1004).

Most patients found the bespoke physiotherapy intervention to be valuable. Those who responded positively appreciated the way it was tailored to their individual condition, and some contrasted it favourably with physiotherapy that they had previously received, which was characterized as ‘tick box physiotherapy’ by one patient (1008).

*The first appointment I had, she sat me down and we went through what problems I had, like day‐to‐day routine. And then she just wanted to see what I could do and then she examined my back and my hip and my leg and everything (1046).*

Patients who valued the physiotherapy appreciated the range of its components, including exercises to reduce pain and discomfort, techniques for improving spinal movement, walking and posture, and manual therapy and cardiovascular exercise.

*I learned to walk again properly, because with having pain on one side, I transferred my weight onto my left side and I was walking with a limp. So I was taught how to stand and walk and use my muscles again properly (1058).*

*There was one exercise that involved me crossing my legs and putting pressure on the opposite knee that really relieved the pain in the bottom of my back (1045).*

Some patients found the counselling element of the physiotherapy, such as pain management advice, helpful in terms of building up their self‐confidence.

*A lot of it was the confidence side of things and the counselling that I got from the physiotherapist that you can bend like this and pick up something quite heavy and also the development of the exercises to get over the psychological side of things (1001).*

A small number of patients did not find the physiotherapy to be of value, reporting that it aggravated the level of pain and discomfort prior to their surgery. One participant, in retrospect, would have preferred to have had the surgery without any physiotherapy:

*I think if you could have that choice, then I would have gone for surgery and just got it over and done with* (1014).

Five of the patients interviewed did not proceed with surgery, reporting that the physiotherapy had either successfully relieved their symptoms, or that the surgery was too risky. In four of these five cases, the patient's consultant and physiotherapist concurred that surgery was not necessary. As can be seen in Table 2, those who decided not to proceed with surgery experienced significant improvements in perceived pain and disability levels, as measured using the Oswestry Disability Index (Fairbank *et al.,* 1980).

#### Patients' perceptions of the value of physiotherapy as an adjunct to surgery

Although 16 patients in the sample elected to proceed with surgery, most believed that the physiotherapy had had some positive benefit prior to surgery. Those who responded positively thought the physiotherapy improved their mobility and strength, which in turn helped them to recover more quickly after their surgery.

*[the physiotherapy helped to keep] the flexibility and my body supple so that as soon as I'd had my surgery, my body was ready to do the exercises. So that can only be a good thing for post‐op recovery (1008).*

Some patients believed that the education received from the physiotherapists ensured that they did not slip into bad habits after their operation:

*[The physiotherapy] has taught me how to get out of bed properly, after the surgery, because they told me how to do it before I had my surgery (1025).*

Other patients spoke of the confidence that the physiotherapy had given them in terms of managing their symptoms post‐surgery*:*

*I have learned a lot from having the physiotherapy and I think it has made me probably stronger mentally, thinking I have had it and it has done me good (1058).*

One patient mentioned that the physiotherapy made him think about improving his own health after his surgery: *It has made me start exercising, which is probably a good thing. The physio has got me into doing exercises on a daily basis* (1045).

Another reported that the physiotherapy removed all pain prior to her surgery*:*

*it got me free of leg pain before the surgery, it works for me, because it got rid of my leg pain. It got rid of all my pains* (1049).

We asked patients if they could say which intervention (the surgery or the physiotherapy) had the most impact on their recovery from sciatica. Most stressed that they found the physiotherapy useful because, although the operation alleviated their leg pain, the physiotherapy helped to alleviate the associated back pain through a combination of manual therapy, exercises and adjustments to posture, walking and movement.

*It is definitely both [interventions working together] without a doubt. The physiotherapy has given me the confidence that I can manage any symptoms… my back feels different from what it did before. It does feel easier, but the surgery has taken away the leg pain…my right leg feels stronger than what it did before the surgery, so it is definitely a bit of both (1001).*

## Discussion

This paper has reported on findings from the nested, qualitative component of a pilot RCT of physiotherapy for patients awaiting lumbar micro‐discectomy surgery. The findings contribute to the current debate about the value of physiotherapy for the treatment of sciatica by presenting patients' own views and experiences about the value of a bespoke physiotherapy intervention — as an intervention in its own right, and as an adjunct to lumbar micro‐discectomy surgery. In addition, we have presented accounts of how sciatica impacted on patients' quality of life before they entered the pilot trial.

From our analysis, patients reported that their sciatica had a profoundly negative impact on their quality of life. Patients we interviewed reported mobility problems, being unable to perform activities of daily living and being unable to work and to undertake leisure activities. Patients also spoke of sleep disruption: this is a common complaint in people with low back pain, with disruption in terms of duration, quality and satisfaction of sleep being identified (Grainne *et al.,* 2011). Patients in our sample also reported feelings of anxiety and depression, which concurs with current research that suggests such mental health issues are associated with neuropathic pain (Uher and Bob, 2013). Taken together, our findings are in accordance with previous literature, which has suggested that sciatica is distinctive in terms of the detrimental impact it can have on quality of life (Andersson, 1999; Waddell, 2004; Webster *et al.,* 2005; Grovle *et al.,* 2010; Ong *et al.,* 2011).

Most patients in the sample did not expect the bespoke physiotherapy intervention to remove the need for lumbar micro‐discectomy surgery; rather, they hoped it would be useful in managing their condition prior to the surgery. However, most patients found the physiotherapy valuable, highlighting exercises to reduce pain and discomfort, techniques for improving spinal movement, walking and posture, manual therapy, cardiovascular exercise and strengthening. This is somewhat at odds with the findings of a systematic review by Searle *et al.* (2015) who found strengthening exercises were beneficial for patients with chronic low back pain but cardiorespiratory exercise not so. These differences may be attributable to the difference in patient populations with the focus of Searle *et al.* (2015) being on the chronic low back pain population and not those suffering specifically with sciatica. For a minority of patients, the physiotherapy was ineffective, with some complaining that it exacerbated or did not reduce the pain and discomfort they were experiencing. Of the 21 patients interviewed, only five elected not to proceed with the surgery after their physiotherapy. Despite this, most of those who underwent surgery believed that the physiotherapy aided their recovery, through improving flexibility and mobility, strengthening their muscles and providing them with practical techniques to manage their condition.

### Strengths and limitations of the study

This qualitative study should be evaluated in terms of its strengths and limitations. Its strengths lie in its rigorous and emergent approach to data collection and analysis, whereby preliminary emergent themes were tested out in subsequent patient interviews. This approach was adopted in part because of the embedded nature of the study design: this qualitative study was nested within a pilot RCT, with a pre‐determined maximum sample size. The second key strength of the study was that the qualitative research team comprised both a non‐clinical health services researcher (J. B.) and a physiotherapist who co‐led the main pilot trial (R. N.). J. B., a non‐physiotherapist, undertook the data collection, in an effort to reduce researcher bias. However, R. N. was involved in interpreting the findings, thus ensuring that data were interpreted by a member of the research team with clinical knowledge of the topic area.

The study has three main limitations. Firstly, and in common with most qualitative studies, it is small in scale and thus does not claim to be generalizable; rather, the study provides insights into the views and experiences of an intervention for a specific condition. Secondly, as the study was undertaken within one National Health Service Trust in the UK, the findings cannot be generalizable to health systems outside this country. Thirdly, due to the study's pre‐determined maximum sample size, saturation of data could not be reached with regard to all the issues in the interview schedule. Although saturation of data concerning patients' views and experiences of the physiotherapy was achieved after approximately 15 interviews, the small number of patients in the intervention arm of the study who did not proceed with surgery (*n* = 5) meant that saturation of data regarding the reasons for this decision could not be reached.

### Implications for further research and physiotherapy practice

In conclusion, the importance of identifying and managing patients with sciatic symptoms in primary care has recently been stressed, including surgical intervention for those cases that are slow to resolve with conservative means such as medication or physiotherapy (Ong *et al.,* 2011). Further work is needed to evaluate if earlier physiotherapy treatment could prevent the need for surgery in a sub‐group of patients with sciatica. We would suggest that further qualitative research should be undertaken into patients' views and experiences of bespoke physiotherapy delivered specifically within primary care settings.

Our findings indicate that many patients with sciatica would welcome the opportunity to receive a bespoke physiotherapy programme prior to lumbar micro‐discectomy surgery. It should be pointed out that patients in our sample may have had an inherent bias against the physiotherapy at the start of the study, due to the positioning of the pilot trial within secondary care, whereby all patients in the intervention arm were on a waiting list for surgery. If this bias was indeed the case, then the mainly positive patient experiences of physiotherapy reported in this paper are particularly encouraging for those who have advocated conservative treatment alongside (or as a possible alternative to) surgery for patients with sciatica.

## Supporting information

Supporting info item

Supporting info item
