# Supplementary material for: Physiotherapy for Patients with Sciatica Awaiting Lumbar Micro‐discectomy Surgery: A Nested, Qualitative Study of Patients' Views and Experiences
Source: Physiother Res Int. 2016 Feb 23;22(3):e1665. doi: 10.1002/pri.1665 (PMC5516132; doi:10.1002/pri.1665)
Supplement: Supplementary file 1 — Supporting info item [file PRI-22-na-s001.doc]

**APPENDIX 1: INTERVIEW SCHEDULES**

**Opening preamble for each interview**

Thank you for agreeing to take part in an interview today with regard to the research study on physiotherapy as a treatment for people with sciatica. We will both need to sign and date the consent form before we proceed with the interview, and with your permission, I would like to record the interview.

1. **Patients that choose not to proceed with surgery after physiotherapy (interviews to take place approximately two weeks after the end of the course of physiotherapy)**
2. Can you tell me first of all for how long you have suffered from sciatica, and what impact it has had on your life before you were placed on the waiting list for surgery?

- *Prompt: has the condition affected your mental health in any way? (Such as anxiety, depression, ability to concentrate?)*
- *Has your condition had any adverse impact on your friends or family?*
- *What prompted you to seek a referral from your GP?*
- *Did you discuss the decision to seek a referral with your immediate family or friends? Did they influence the decision to seek a referral?*
- *Do you think you were referred to the consultant promptly by your GP?*
- *Do you feel there was any unncessary delay in getting the referral?*

1. Can you tell me why you agreed to take part in the study?

- *Prompt: Was it your belief that you could have the surgery sooner if you took part in the study?*

1. As you know, you received a course of physiotherapy treatment while awaiting surgery. I would like you to tell me what your expectations were of physiotherapy before you started the course of treatment. In other words, what was your goal in having the physiotherapy?

- *Prompt: (1) to get back to work; (2) to reduce pain; (3) to enable the pursuit of leisure activities (4); to enable the person to play with their children or grandchildren; (5) to improve sleeping patterns; (6) to improve psychological wellbeing; (7) to prevent the need for surgery*

1. Did the physiotherapy treatment meet your expectations and your goal in having the treatment?

- *Prompt: explore the reasons for whether the physiotherapy exceeded or otherwise the patient’s expectations and goal*

1. Did you have any concerns or anxieties about the physiotherapy prior to the start of the treatment?

- *Prompt: explore the reasons for these concerns or anxieties*

1. Did you feel optimistic about the physiotherapy treatment?

- *Prompt: explore the reasons for feelings of optimism or pessimism*

1. What aspects of the phsiotherapy did you feel were the most helpful?

- *Prompts: advice and education, pain management advice, manual ‘hands on’ therapy, exercise therapy and rehabilitation, appointment times, convenience of the parking at clinic, how the appointment was scheduled*

1. What aspects of the physiotherapy did you feel were the least helpful?
2. As you know you received a session of physiotherapy while you were in the study once a week? What are your thoughts on the intensity of the physiotherapy? Were you happy to have one session a week or would have preferred to have more sessions a week?

- *Prompts: advice and education, pain management advice, manual ‘hands on’ therapy, exercise therapy and rehabilitation, appointment times, convenience of the parking at clinic, how the appointment was scheduled*

1. Did you have any physiotherapy treatment before seeing the consultant?

- (If yes), Was the physiotherapy you received before seeing the consultant different to the physiotherapy you had while awaiting surgery?
- (If yes) How was it different?
- *Prompts: advice and education, pain management advice, manual ‘hands on’ therapy, exercise therapy and rehabilitation*

1. Do you think it would have been beneficial to have received the targeted type of phyiotherapy that you received during this research earlier (i.e. before being referred for scanning and surgery?

- *Prompts*: explore the reasons for or against

1. What specific physiotherapy did you have?

*Prompts:*

- - Did your treatment relate to a specific goal
  - Did it involve exercises? If so can you remember what type? (on your stomach, on your back, standing, any arching backwards execises, core stability exercises (usually static exercises lying on back to strengthen stomach muscles))
  - Did it involve any hands on therapy?

1. Was it made clear to you what the exercises were designed to achieve?
   - For example, to get you back to work/sport or something more specific like stretching your back?
2. Did you have any concerns or anxieties about the possibility of surgery while you were on the waiting list for surgery?

- *Prompt: explore the reasons for these concerns or anxieties*
- *Did you do any of your own research into the procedure? If so, what did you find out? Did this make you more or less anxious about the procedure?*

1. As you know, you are not proceeding with surgery following the course of physiotherapy. Was the decision not to proceed mainly your decision, or that of your consultant, or was the decision reached jointly?
2. Please can you describe for me your reasons for not proceeding with the surgery?

*Prompts:*

- - Feeling better
  - Can cope with the symptoms as they are at present
  - Consultant decided not to progress with surgery
  - Other

1. Could you tell me about any previous thoughts or experiences you may have had about physiotherapy before taking part in this research study?
2. Do you have any concerns about your back problem in the future?

- *Prompt: explore the reasons for these concerns or anxieties*

1. Do you feel you have been given enough help to manage your symptoms in the future?

- *Prompt: explore the nature and reasons for any unmet needs*

1. Are you optimistic about your future health having undergone the course of physiotherapy?

- *Prompt: explore the reasons for any reasons given for cause for optimism or pessimism*

1. Do you feel you have received any unncecessary treatment?
2. Is there any thing further you want to say about your physiotherapy treatment that we have not already covered in this interview?

***Close of interview***: thank you very much for participating in this interview. A copy of the transcript will be sent to you for your information

1. Patients that choose to proceed with surgery (interviews to take place 2 weeks post surgery)
2. Can you tell me first of all for how long you have suffered from sciatica, and what impact it has had on your life before you were placed on the waiting list for surgery?

- *Prompt: has the condition affected your mental health in any way? (Such as anxiety, depression, ability to concentrate?)*
- *Has your condition had any adverse impact on your friends or family?*
- *What prompted you to seek a referral from your GP?*
- *Did you discuss the decision to seek a referral with your immediate family or friends? Did they influence the decision to seek a referral?*
- *Do you think you were referred to the consultant promptly by your GP?*
- *Do you feel there was any unncessary delay in getting the referral?*

1. Can you tell me why you agreed to take part in the study?

- *Prompt: was it your belief that you could have the surgery sooner if you took part in the study?*

1. As you know, you received a course of physiotherapy treatment while awaiting surgery. I would like you to tell me what your expectations were of physiotherapy before you started the course of treatment. In other words, what was your goal in having the physiotherapy?

- *Prompt: (1) to get back to work; (2) to reduce pain; (3) to enable the pursuit of leisure activities (4); to enable the person to play with their children or grandchildren; (5) to improve sleeping patterns; (6) to improve psychological wellbeing; (7) to prevent the need for surgery*

1. Did the physiotherapy treatment meet your expectations and your goal in having the treatment?

- *Prompt: explore the reasons for whether the physiotherapy exceeded or otherwise the patient’s expectations and goal*

1. Did you have any concerns or anxieties about the physiotherapy prior to the start of the treatment?

- *Prompt: explore the reasons for these concerns or anxieties*

1. Did you feel optimistic about the physiotherapy treatment?

- *Prompt: explore the reasons for feelings of optimism or pessimism*

1. What aspects of the phsiotherapy did you feel were the most helpful?

- *Prompts: advice and education, pain management advice, manual ‘hands on’ therapy, exercise therapy and rehabilitation*

1. What aspects of the physiotherapy did you feel were the least helpful?

- *Prompts: advice and education, pain management advice, manual ‘hands on’ therapy, exercise therapy and rehabilitation*

1. As you know you received a session of physiotherapy while you were in the study once a week? What are your thoughts on the intensity of the physiotherapy? Were you happy to have one session a week or would have preferred to have more sessions a week?
2. Did you have any physiotherapy treatment before seeing the consultant?

- (If yes), Was the physiotherapy you received before seeing the consultant different to the physiotherapy you had while awaiting surgery?
- (If yes) How was it different?
- *Prompts: advice and education, pain management advice, manual ‘hands on’ therapy, exercise therapy and rehabilitation*

1. Do you think it would have been beneficial to have received the targeted type of phyiotherapy that you received during this research earlier (i.e. before being referred for scanning and surgery?

- *Prompts*: explore the reasons for or against

1. What specific physiotherapy did you have?

Prompts:

- - Did your treatment relate to a specific goal
  - Did it involve exercises? If so can you remember what type? (on your stomach, on your back, standing, any arching backwards execises, core stability exercises - usually static exercises lying on back to strengthen stomach muscles)
  - Did it involve any hands on therapy?

1. Was it made clear to you what the exercises were designed to achieve?

- *Prompts:* For example, to get you back to work/sport or something more specific like stretching your back?

1. Did you have any concerns or anxieties about the possibility of surgery while you were on the waiting list for surgery?

- *Prompt: explore the reasons for these concerns or anxieties*
- *Did you do any of your own research into the procedure? If so, what did you find out? Did this make you more or less anxious about the procedure?*

1. Could you tell me about any your previous thoughts or experiences you may have had about physiotherapy before taking part in this research study?
2. Having gone through the surgery for your sciatica, do you feel it was beneficial to have had physiotherapy first?

- *Prompts: explore any reasons given for or against*

1. Do you feel the surgery has lessened the pain in your leg and your back?

- *Prompts: explore any reasons given for lessening of pain in on place rather than the other*

1. Have you noticed after having the surgery that the discomfort in your back has lessened or that the nature of the discomfort has changed at all?

- *Prompt: post-surgery have you noticed the discomfort in your back changing from a pain to an ache?*

1. Do you feel the physiotherapy has made any difference to your condition?

- *Prompts: explore any reasons given for or against*

1. What did you think the surgery could achieve for you over and above the physiotherapy?

- *Prompt:* extent to which the person agrees and understands that surgery is to ease leg pain, and does not treat the back

1. Do you have any concerns about your back problem in the future?

- *Prompt: explore the reasons for these concerns or anxieties*

1. Do you feel the in-patient physiotherapy that you received while you were in hospital was useful to you?

- *Prompt: Did you receive sufficient information about what you can and can’t do after your operation?*
- *What would you have liked more information about, if anything?*

1. Do you feel it is beneficial to have post-operative physiotherapy 2 weeks after surgery or would you prefer instead to wait to see the consultant 8 weeks after surgery with no physiotherapy in the meantime?

- *Prompts: explore perceived usefulness of post-surgery exercises and early contact with physiotherapist or if 8 weeks of rest is more useful*

1. Do you feel you have been given enough help to manage your symptoms in the future?

- *Prompt: explore the nature and reasons for any unmet needs*

1. Are you optimistic about your future health having undergone the course of physiotherapy and the surgery?

- *Prompt: explore the reasons for any reasons given for cause for optimism or pessimism and ask if it is the physiotherapy and/or the surgery that gives most cause for optimism or pessimism*

1. Do you feel you have received any unncecessary treatment?
2. Is there any thing further you want to say about your physiotherapy treatment or the surgery that we have not already covered in this interview?

***Close of interview***: thank you very much for participating in this interview. A copy of the transcript will be sent to you for your information
