# Supplementary material for: Physiotherapy for Patients with Sciatica Awaiting Lumbar Micro‐discectomy Surgery: A Nested, Qualitative Study of Patients' Views and Experiences
Source: Physiother Res Int. 2016 Feb 23;22(3):e1665. doi: 10.1002/pri.1665 (PMC5516132; doi:10.1002/pri.1665)
Supplement: Supplementary file 2 — Supporting info item [file PRI-22-na-s002.docx]

**Appendix 2 Example of the utilisation of the physiotherapy approach**

| **Assessment item** | **Intervention** |
| --- | --- |
| **Neurological Findings**  Positive SLR 40 otherwise normal reflexes, power and sensation. | Encouragement of ‘normal’ neural health by using the spine as normally as possible thus ensuring mobilisation of neural (and other) tissues in a functional context relevant to the individual. In this case bending down to feed the cats in the morning. |
| **Psychological barriers to recovery**  Fear of physiotherapy. Previous physiotherapy, for this episode made the pain significantly worse.  Kinesiophobia, particularly into lumbar flexion. This was probably secondary to poor education on the normal kinematics of the disc and the patients fear of it ‘popping out’. | Primarily the management of this element was directed at reasoning, through education of ‘normal’ anatomy and pathology as to why the previous physiotherapy may have been painful. The logical sequence of this approach then took the patient through why it would be beneficial to move and use their spine as normally as possible. The end-stage of this was to show the patient, that by moving ‘normally’ and without fear they could achieve a (reasonable) functional goal such as putting on shoes or feeding the cats. |
| **Movement restriction**  Minimal lumbar flexion due to both fear and joint/muscular restriction. | Mobilisation of restricted areas to increase movement, decrease fear and pain. This is then promoted through behavioural change by using the movement gained in a functional arena. For example putting on shoe by bending rather than asking someone else to do it. |
| **Conditioning**  Prior to the onset of LRS the patient was a keen runner and gym-goer. The patient declares that they feel ‘unfit’ and miss both the physical and social elements of attending the gym. | The key message to the patient is that it is safe (education) to exercise in the absence of red-flag symptoms/signs. This message was promoted by the introduction of a gym programme (supervised by the physiotherapist). Although the patient was unable to run due to exacerbation of pain, they were able to cycle and perform other exercises. |
| **Movement control**  Sub-optimal control of lumbar spine during gait in the sagittal plane, exemplified by the increase in hyperlordosis on heel-strike and tibial progression on the left leg. | Movement re-education; concentrating on the functional difficulty the patient was declaring: walking. Progression of sagittal control of the lumbar spine during gait, particularly avoiding hyper-extension during initial phases of gait. |
| **Pain**  Pain levels were around 5-7 on the VAS score and the patient thought that the pain was poorly controlled. | A review of the analgesia was undertaken with the patient. It was identified that they were taking analgesia when the pain become unbearable rather than prophylactically. They were encouraged to take the medication as per their prescribers instructions. The patient was also asked to see their G.P with regards to the possible commencement of anti-neuropathic pain medication. |
| **Advice & Education**  Faulty cognitions such as the patient thought ther disc would ‘pop out’ if she moved her Lumbar spine. | Education on ‘normal’ disc make-up, aetiology of disc prolapse and discussion on prognosis. This included using adjuncts such as websites, models and bringing in some evidence from contemporary research. The focus on this approach is on the patient needs and attempting to make it discursive, non-jargon and not didactic. The primary aim is to ensure that , excluding red-flag symptoms/signs that it is safe to exercise and got on with life as normally as possible. |
